# Risk factors for pleural effusion in children with plastic bronchitis caused by pneumonia

**DOI:** 10.3389/fped.2024.1412729

**Published:** 2024-10-24

**Authors:** Xiaoliang Lin, Enhui Xu, Tan Zhang, Qiguo Zhu, Deyi Zhuang

**Affiliations:** ^1^ Department of Respiratory Medicine, Xiamen Children’s Hospital (Children’s Hospital of Fudan University at Xiamen), Xiamen, China; ^2^ Fujian Key Laboratory of Neonatal Diseases, Xiamen Children's Hospital (Children's Hospital of Fudan University at Xiamen), Xiamen, China

**Keywords:** plastic bronchitis, pleural effusion, clinical characteristics, risk factors, children, inflammation

## Abstract

**Objective:**

We aimed to investigate the clinical features of children with plastic bronchitis caused by pneumonia, and assess the risk factors for pleural effusion (PE) in plastic bronchitis.

**Methods:**

A retrospective study was conducted on children with plastic bronchitis and hospitalized in Xiamen Children’s Hospital from January 2017 to April 2023. Participants were categorized based on the presence of PE. Their clinical manifestations and laboratory findings were analyzed.

**Results:**

Sixty-nine children with plastic bronchitis were enrolled: 34 cases (49.27%) in the non-PE group and 35 cases (50.72%) in the PE group. No significant differences were found in sex, age, and etiology between the two groups. Significant differences were found in fever duration, C-reactive protein (CRP), albumin and lactate dehydrogenase (LDH) (p<0.05). A receiver operating characteristic (ROC) curve analysis showed that the cut-off values for higher risk of PE were CRP >31.66 mg/L or LDH >551 U/L. The prediction of PE was performed with the combination of CRP >31.66 mg/L and LDH >551 U/L, using multivariate logistic regression analysis. The area under the curve (AUC) for logistic regression was 0.797. Elevated CRP increased the risk of PE (odds ratio [OR] 8.358, 95% confidence interval [CI] 2.179–42.900, p=0.0042), elevated LDH increased the risk of PE (OR 5.851 [95% CI 1.950–19.350], p=0.0023).

**Conclusion:**

The combined detection of CRP and LDH helps predict the risk of PE in children with plastic bronchitis caused by pneumonia.

## 1 Introduction

Plastic bronchitis (PB) refers to the formation of a local or extensive “dendritic” cast in the airway, leading to partial or complete obstruction of the airway, which could be visualized with bronchoscopy. PB was more common in children than in adults, the prevalence was estimated to be 6.8 per 100 000 children (1). Pediatric PB was previously believed to develop mainly in children with congenital heart disease, i.e., in 4%–14% of postsurgical patients (2). Recent studies drew attention to infection-induced PB (3). The mortality of PB was as high as 29% in patients with cardiac defects (4), and 3.6% in infection-induced PB (5). Misdiagnosis of PB is common due to its acute onset, rapid progression, and lack of typical imaging features (6). Therefore, a timely diagnosis and removal of the plastic phlegm plug are crucial to improve the prognosis.

Pneumonia-induced PB was reported mainly in those infected with *Mycoplasma pneumoniae* pneumonia (MPP), adenovirus, and influenza virus (7–9). Infection triggers airway inflammation, which can lead to hyperviscous mucus in the airways, accumulation of bronchial epithelial cells, and deficiency of mucociliary clearance in the bronchus (10). Meanwhile, pleural effusion (PE) can occur due to a strong inflammatory response, in which proinflammatory mediators increase vascular permeability and thicken the parietal pleura (11). PE was identified in 19% of adults hospitalized with pneumonia (12). Two thirds of children with PE had a volume less than 1/4 of the thorax (13). Nonetheless, PE alone, regardless of size, signifies twice higher 30-day mortality in patients with pneumonia (14). Moreover, PE causes lung compression and restrictive ventilation dysfunction, and was proven to be related to PB in patients with MPP (15). The complication of PE in patients with PB could aggravate impaired respiratory function. Therefore, PE should be closely monitored in patients with PB, and managed with caution.

Biochemical markers were reported to predict PE or PB in children with pneumonia. For example, elevated serum C-reactive protein (CRP), lactate dehydrogenase (LDH), aspartate transaminase (AST), and D-dimer were found to be related to PE in children with MPP (16, 17). In addition, PE was more common in refractory MPP than in general MPP, and risk factors for refractory MPP included serum CRP, LDH, D-dimer, and ferritin (13, 18–20). Meanwhile, PB in MPP could be predicted by CRP (21), as well as LDH (7, 21–23). Proinflammatory cytokines interleukin-6 and interleukin-8 were elevated in patients with PB as well (7, 22, 23). The elevated inflammatory markers mentioned above indicate strong inflammatory responses in patients with PB and PE. Nonetheless, no biomarkers are yet available to predict PE in patients with PB.

We hypothesized that inflammatory-related factors predict PE in children with PB, and focused on laboratory tests that are commonly available. We retrospectively analyzed the clinical characteristics of hospitalized children with PB at Xiamen Children’s Hospital. We categorized participants into PE and non-PE groups, analyzed their hematological and inflammatory indicators. We hope to aid in the diagnosis and treatment of PE and PB in patients with pneumonia.

## 2 Methods

### 2.1 Data collection

We retrospectively collected data from children aged 1 month to 18 years, who were diagnosed with pneumonia and hospitalized at Xiamen Children’s Hospital from January 2017 to April 2023. We included those patients diagnosed with PB by bronchoscopy, specifically, patients who showed partial or total airway obstruction and dendritic bronchial cast through bronchoscopy. For patients who received more than one bronchoscopy, we exclusively included data from the first bronchoscopy. We excluded patients with incomplete clinical data. The diagnosis of PE was based on chest computed tomography (CT) examinations.

We collected clinical data, including age, sex, underlying diseases, nutritional status, and duration of fever. We collected hematological indicators (white blood count, neutrophil, lymphocyte, and platelet), metabolic panel (albumin and LDH), inflammatory markers (CRP, procalcitonin, and erythrocyte sedimentation rate), coagulation function (D-dimer, fibrinogen, and fibrinolytic product), and images of chest CT scan. Serum CRP was tested with the high-sensitivity CRP turbidimetric immunoassay kit (Lifotronic Technology, China). Serum PCT was measured using the BRAHMS-PCT kit (Thermo Fisher Scientific, USA). ESR was determined with TEST1/THL analysis system (Alifax, Italy). The coagulation function was tested with CS-5100 analyzer (Sysmex, JAPAN).

Pathogenic diagnosis was based on serum anti-*Mycoplasma pneumoniae* immunoglobulin M (MP-IgM), microbial culture using bronchoalveolar lavage fluid (BALF), and detection of respiratory viruses using BALF. To detect respiratory viruses, before January 2022, respiratory virus antigens were detected by direct immunofluorescence assay kits (Diagnostic Hybrids, Inc., San Diego, CA), including influenzae viruses A and B, parainfluenza viruses 1, 2, and 3, respiratory syncytial virus, and adenovirus. From January 2022 onwards, respiratory viruses were detected with nucleic acid amplification tests, using the SureX 13 respiratory pathogen multiplex detection kit (Ningbo HEALTH Gene Technologies, China) according to the manufacturer’s instructions. Nucleic acid-based tests included influenza A virus (RNA), influenza A 2009 (H1N1) virus (RNA), seasonal influenza A (H3N2) virus (RNA), influenza B virus (RNA), parainfluenza virus (RNA), coronavirus (RNA), respiratory syncytial virus (RNA), rhinovirus (RNA), bocavirus (DNA), human metapneumovirus (RNA), adenovirus (DNA), MP (DNA) and chlamydia (DNA).

Clinical data were collected at the time of admission. Blood tests were performed within 24 h before admission or within 2 h after admission. CT examinations were performed before admission or within 3 days after admission. Bronchoscopy was performed within 3 days after admission.

### 2.2 Ethics statement

This study was approved by the Ethics Committee of Xiamen Children’s Hospital (protocol code 2024 [04] and date of approval 2024-02-27). Guardians of all participants gave written informed consent. All procedures followed the Helsinki Declaration revised in 2013, and institutional and national ethical standards.

### 2.3 Statistical analysis

SPSS software (version 22.0; SPSS Inc, Chicago, IL, USA) was used for data analysis. Based on the presence or absence of PE on CT images, patients were categorized into PE group and non-PE group. Continuous variables with normal distribution were given as mean ± standard deviation (SD), and compared by unpaired t test; categorical variables as numbers and percentages (%), and compared using the chi-squared test. The risk factors for PE were analyzed with multivariate logistic regression analysis, interpreted with odds ratio (OR) and confidence interval (CI). The prediction efficiency was analyzed with the receiver operating characteristic (ROC) curve and area under the curve (AUC). A two-sided p value less than 0.05 was considered statistically significant.

## 3 Results

### 3.1 Demographic and clinical features of participants: PE vs. non-PE group

In total, 69 patients were included in this study (Table 1). Among them, 35 cases were diagnosed with PE and defined as the “PE group”, the other 34 cases were defined as the “non-PE group”. The age of onset ranged from 3 months to 13 years and 1 month, and the mean age was 54.97±36.08 months. The mean age between the PE and non-PE group was not significantly different (50.80±38.29 months vs. 59.26±33.68 months, unpaired t test, p=0.336). Four patients had underlying diseases, all of them were in the non-PE group (2 cases of bronchial asthma and 2 cases of epilepsy). No significant differences were found between the two groups in sex, underlying disease, and nutritional status. The duration of the fever was significantly different between the two groups ($\chi^{2}=6.155$, p=0.048). The ratio of patients with prolonged fever (≥1 week) was higher in the PE group than in the non-PE group (29/35 vs. 19/34). Regarding treatment, methylprednisolone was prescribed in 56 of the 69 cases.

**Table 1:** Demographic, clinical features of patients with PB: PE vs. non-PE group.

Variables	PE group (n=35)	Non-PE group (n=34)	$\chi^{2}$	p value
Sex				
Male	20	18	0.123	0.726
Female	15	16		
Underlying diseases				
Yes	0	4	4.371	0.054
No	35	30		
Nutritional status				
Normal	32	26	2.880	0.090
Malnutrition	3	8		
Fever duration				
No fever	1	5	6.155	**0.048**
<1 week	5	10		
≥1 week	29	19		

Note: PB, plastic bronchitis; PE, pleural effusion. The p value with statistical significance is highlighted with bold font.

**Figure 1:**
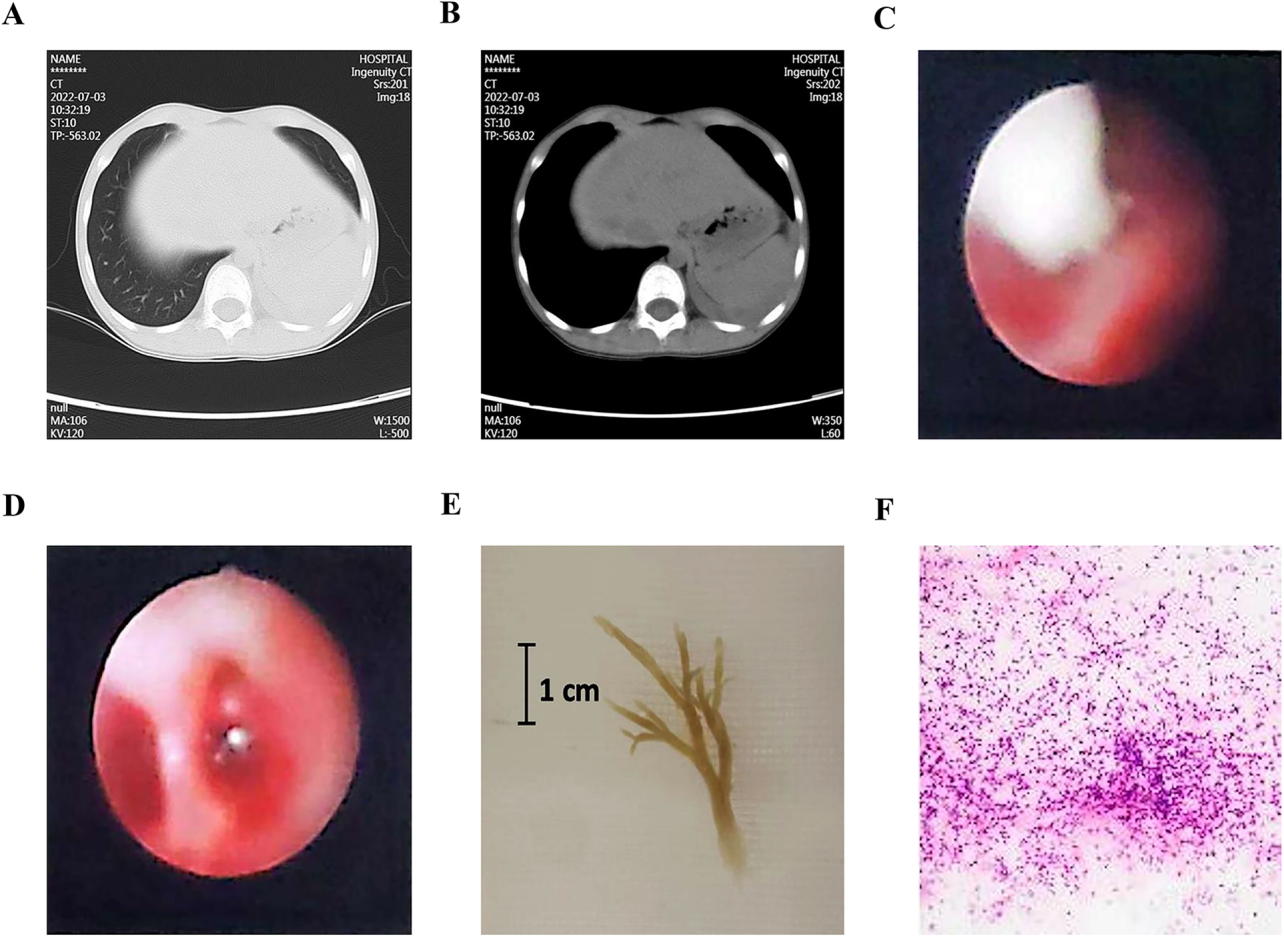
A representative case with plastic bronchitis and pleural effusion. **(A,B)** Chest CT images showed an obstruction of the proximal bronchi in the left lower lobe, atelectasis and consolidation, and pleural effusion. **(C)** Bronchoscopy noticed a white mucus plug. **(D)** After the removal of the plug, congestive and swelling mucosa were observed. **(E)** A cast of plastic bronchitis was removed. **(F)** Cytopathology of bronchoalveolar lavage fluid revealed 60% neutrophils, 30% phagocytes, and 10% ciliated columnar epithelial cells.

A representative case with PB and PE is demonstrated in Figure 1. This case is a 7-year-old boy, had fever for five days, and examined with chest CT scan on the third day of admission. His chest CT images showed obstruction of the proximal bronchi in the left lower lobe, atelectasis and consolidation in the left lower lobe of the lung, and a small amount of pleural effusion (Figures 1A,B). Interventional bronchoscopy was performed on the third day of admission. A white mucus plug was noticed obstructing the left lower lobe (Figure 1C). After removing the mucus plug, we observed congestive and swelling mucosa (Figure 1D). A cast of plastic bronchitis with approximately 3 cm in length was removed (Figure 1E). The cytopathology of BALF revealed 60% neutrophils, 30% phagocytes, and 10% ciliated columnar epithelial cells (Figure 1F). Nucleic acid-based pathogen detection using BALF detected *Mycoplasma pneumoniae* (MP) and human metapneumovirus.

### 3.2 Etiological analysis: PE vs. non-PE group

Pathogenic analysis was performed for all participants (Table 2). To detect respiratory viruses, 60 patients were tested for antigens, 9 patients were tested by nucleic acid-based tests. One or more pathogens were detected in most patients (56/69). MP was the most common pathogen, detected in 24 cases without co-infected pathogens, in 7 cases with adenovirus co-infection, and in 2 cases with human metapneumovirus. All 14 cases with exclusive viral infection were detected with adenovirus. In patients with mixed infections, adenovirus was the most common pathogen, detected in 7 cases with MP co-infection, and in 5 cases with a co-infection of bacteria or fungus.

**Table 2:** Etiology of of patients with PB: PE vs. non-PE group.

Variables	PE group (n=35)	Non-PE group (n=34)
MP	12	12
Viral	9	5
ADV	9	5
Bacterial	0	2
Co-infection	6	10
ADV + MP	2	3
ADV + bacteria	2	1
ADV + fungus	0	2
MP + metapneumovirus	1	1
MP + ADV + parainfluenza virus 3	1	0
MP + ADV + aspergillus flavus	0	1
Parainfluenza virus + Bocavirus	0	1
RSV + *Streptococcus pneumoniae*	0	1
Negative	8	5

Note: PB, plastic bronchitis; PE, pleural effusion; MP, *Mycoplasma pneumoniae*; ADV, adenovirus; RSV, respiratory syncytial virus.

### 3.3 Laboratory findings: PE vs. non-PE group

We compared hematological indicators, metabolic panel, inflammatory markers, and coagulation function between the PE and non-PE group (Table 3). Among these indicators, CRP was significantly higher in PE group than in non-PE group (34.11±32.87 mg/L vs. 18.69±26.41 mg/L, unpaired t test, p=0.036). Albumin was significantly lower in PE group than in non-PE group (34.80±5.13 g/L vs. 37.75±4.29 g/L, unpaired t test, p=0.012). LDH was significantly higher in PE group than in non-PE group (933.31±737.99 U/L vs. 507.6±252.27 U/L, unpaired t test, p=0.002). The other indicators did not show significant differences between the two groups.

**Table 3:** Laboratory findings of patients with PB: PE vs. non-PE group.

Variables	PE group	Non-PE group	t value	p value
WBC ($\times 10^{9}$/L)	8.02±5.92	7.26±3.46	0.651	0.518
N (%)	75.65±84.89	58.99±17.54	1.121	0.266
L (%)	31.70±13.05	31.40±15.25	0.090	0.929
PLT ($\times 10^{9}$/L)	226.29±113.15	231.82±105.24	−0.208	0.836
CRP (mg/L)	34.11±32.87	18.69±26.41	2.144	**0.036**
Albumin (g/L)	34.80±5.13	37.75±4.29	−2.584	**0.012**
LDH (U/L)	933.31±737.99	507.6±252.27	3.224	**0.002**
D-dimer (mg/L)	1.44±1.13	1.01±1.35	1.442	0.154
FIB (g/L)	3.20±0.95	3.31±0.97	−0.501	0.618
FDP (μg/ml)	8.55±6.38	6.30±7.76	1.320	0.191
ESR (mm/h)	33.63±17.15	36.04±23.17	−0.408	0.685
PCT (ng/ml)	2.65±4.68	1.50±3.16	1.193	0.237

Note: PB, plastic bronchitis; PE, pleural effusion; WBC, blood white blood count; N, neutrophil; L, lymphocyte; PLT, platelet; CRP, C-reactive protein; LDH, lactate dehydrogenase; FIB, fibrinogen; FDP, fibrinolytic product; ESR, erythrocyte sedimentation rate; PCT, procalcitonin. The p values with statistical significance are highlighted with bold font.

### 3.4 Multivariate logistic regression analysis of risk factors for PE

Four factors (fever duration, CRP, albumin, and LDH) were proven to be significantly different between the PE and non-PE group, according to previous analyses (Tables 1, 3). Subsequently, we performed a multivariate logistic regression analysis for these four factors to identify potential risk factors for PE in PB patients (Table 4). The duration of the fever was categorized into two groups with a cut-off of 1 week. Among these four factors, the prediction using CRP and LDH showed the highest statistical significance. Elevation in CRP increased the risk of PE (OR 1.020 [95% CI 1.001–1.044], p=0.055). Likewise, elevation of LDH increased the risk of PE (OR 1.002 [95% CI 1.000–1.005], p=0.049). To identify potential predictors, we chose CRP and LDH for a further prediction model, with a liberal cutoff of p<0.10 (24, 25).

**Table 4:** Multivariate logistic regression analysis of risk factors for PE.

Variables	Coefficient	OR (95% CI)	p value
Fever >1 week	0.355	1.427 (0.382–5.471)	0.596
CRP (mg/L)	0.020	1.020 (1.001–1.044)	0.055
Albumin (g/L)	0.006	1.006 (0.863–1.179)	0.937
LDH (U/L)	0.002	1.002 (1.000–1.005)	0.049

Note: PE, pleural effusion; OR, odds ratio; CI, confidence interval; CRP, C-reactive protein; LDH, lactate dehydrogenase.

### 3.5 Prediction of PE using CRP and LDH

In the next step, to evaluate the performance of CRP and LDH in the prediction of PE in patients with PB, we plotted the ROC curves for CRP and LDH individually (Table 5). The AUC of CRP for the prediction of PE was 0.656. The cutoff value of CRP with the maximum Youden index was >31.66 mg/L. The AUC of LDH was 0.716, the best cut-off value of LDH was >551 U/L. We further predicted PE with the combination of CRP >31.66 mg/L and LDH >551 U/L using binary logistic regression analysis. The AUC for logistic regression using the combined CRP and LDH cut-off values was 0.797 (95% CI 0.691–0.904, p<0.0001). With the cut-off of 31.66 mg/L, an elevated CRP increased the risk of PE (coefficient 2.123, OR 8.358 [95% CI 2.179–42.900], p=0.0042). With the cut-off of 551 U/L, an elevated LDH increased the risk of PE (coefficient 1.767, OR 5.851 [95% CI 1.950–19.350], p=0.0023).

**Table 5:** ROC analysis of CRP and LDH in predicting PE.

Variables	AUC (95% CI)	Z	p value	Youden index	Cut-off value	Sensitivity (%)	Specificity (%)
CRP (mg/L)	0.656 (0.532–0.767)	2.304	0.021	0.369	>31.66	45.7	91.2
LDH (U/L)	0.716 (0.595–0.818)	3.422	0.001	0.421	>551.00	68.6	73.5

Note: ROC, receiver operating characteristic curve; CRP, C-reactive protein; LDH, lactate dehydrogenase; PE, pleural effusion; AUC, area under the curve; CI, confidence interval.

## 4 Discussion

We reported a cohort of children with infection-induced PB, and half of the participants were accompanied by PE. An elevation of CRP or LDH increased the risk of PE in children with PB. We established a simple model to predict PE in children with PB, using the combination of CRP >31.66 mg/L and LDH >551 U/L as predictors.

The elevated levels of CRP and LDH in our patients with PE are consistent with previous studies. For example, in children with MPP, those with PE had higher CRP, LDH, and AST (16). Compared to general MPP, PE was more common in refractory MPP, which was predicted by CRP (13, 18, 19), LDH (13, 18–20), and D-dimer (17). CRP is the most common inflammatory marker. LDH can be released from cytoplasm into blood during severe inflammation, indicating an increased permeability of the liver cell membrane. In our study, the combination of CRP and LDH provided the best AUC than individual CRP or LDH, indicating a strong inflammation in patients accompanied by PE. Specifically, we speculate that those patients with PE suffered from an even stronger inflammation than those without PE.

We observed a high prevalence (roughly half) of PE in patients with PB. This incidence of PE is much higher than in patients with pneumonia, among whom 14%–19% were accompanied by PE (12, 14). This prevalence we observed is similar to previous studies. For example, in children with refractory MPP, PE was diagnosed in 39%–50% of patients with PB, compared to 21%–26% of patients without PB (7, 21, 23). In 300 cases of children with influenza virus pneumonia, PE was diagnosed in 53% of patients with PB, compared to 34% of patients without PB (9).

The prevalence of PE may be due to a strong inflammatory response in PB. PB was found to be associated with abnormal pulmonary lymphatic flow in children with congenital heart disease (26). Palyga-Bysiecka et.al proposed that during an infection, the activated mast cells in lung induces lymphangiogenic factors, which cause distension of lymphatic vessels, leading to PB (3, 27, 28). The role of inflammation in the formation of PB can be supported by the cytophathology of BALF from patients in our cohort, which showed mainly inflammatory cells, as presented in Figure 1. Clinically, multiple inflammatory markers were revealed to increase in children with PB. For example, CRP (21), as well as LDH (7, 21–23), were risk factors for PB in MPP. LDH also predicted PB in children with influenza virus pneumonia (9). Other inflammatory markers, such as interleukin-6 and interleukin-8, were found to be higher in patients with PB (7, 22, 23). In our study, CRP, LDH, and erythrocyte sedimentation rate were elevated in both the PE and non-PE groups (Table 3), supporting the role of inflammation in PB.

The high incidence of PE in our study may be related to the prevalence of MP (33/69) and adenovirus (26/69), which were reported to induce strong immune responses. PB was common in MP infection, since MP stimulates the secretion and accumulation of airway mucus, causes abnormalities in the ciliary ultrastructure, leading to mucous plugs (10). Likewise, PB has been reported in adenovirus infection, and may be caused by excessive damage to airway epithelial cells (29). Consistent with our findings, in previous studies of children with PB, 44%–72% had PE, and 62%–77% were infected with MP or/and adenovirus(5, 30). In 45 cases of infection-related PB, 53% were infected with MP, among them 43% had PE (31).

Current management of infection-induced PB includes mainly removal of mucus plugs by interventional bronchoscopy, and concurrent anti-inflammatory treatment with glucocorticoids. Corticoids were effective and safe in treating PB with hemoptysis, and facilitated the relief of PE (32, 33). In clinical practice, some patients with PB needed more than one bronchoscopy to alleviate the obstruction, due to the severity of the obstruction and the continued inflammation. Based on the proposal of this study that both PB and PE are potentially related to a strong immune response, we emphasize anti-inflammatory treatment in patients with PB. The alleviation of immune response may reduce the potential risks of PE, and reduce the risk of multiple bronchoscopy to avoid potential complications.

This study has several limitations. The sample size is limited by the low incidence of PB. Multicenter studies in the future with larger sample sizes could increase statistical power, and investigate PB caused by different pathogens individually. In addition, some of our participants received antibiotic treatment prior to admission, which may affect the results of blood tests. Further studies with a uniform time course and treatment can be designed to control such variations.

In conclusion, we presented a cohort of children with PB in the absence of congenital heart disease, and brought attention to surveillance of PB in pneumonia. PE is common in patients with PB caused by pneumonia. A prediction model with combined CRP >31.66 mg/L and LDH >551 U/L performed well in predicting PE. For a timely diagnosis of PE, since PE may originate from strong inflammation responses, inflammatory markers should be closely monitored in patients with PB. For the treatment of PB, we recommend prescribing anti-inflammatory reagents in addition to interventional bronchoscopy, in hopes of limiting inflammatory responses and therefore reducing the risk of PE. The optimal duration and dose of anti-inflammatory reagents require future clinical trials.

## Data Availability

The original contributions presented in the study are included in the article, further inquiries can be directed to the corresponding author.
